# Acute Pancreatitis Complicating a Case of Dengue Fever: Double Trouble

**DOI:** 10.7759/cureus.19523

**Published:** 2021-11-13

**Authors:** Srinivas Naik, Satish Mahajan, Dhruv Talwar, Gaurav Jagtap

**Affiliations:** 1 Department of Medicine, Jawaharlal Nehru Medical College, Datta Meghe Institute of Medical Sciences (Deemed to be University), Wardha, IND

**Keywords:** flaviviridae, breakbone fever, tropical disease, acute pancreatitis, dengue fever

## Abstract

Dengue has emerged as an alarming concern exhausting the already tired healthcare professionals during the ongoing pandemic of COVID-19. There has been an epidemic of dengue fever with massive underreporting; this might be a result of limited resources as well as the inability to reach healthcare facilities as a consequence of reluctance seen in patients due to the scare of COVID-19. Acute pain in the abdomen has been an alarming sign of dengue; however, its association with acute pancreatitis is rare. We report a case of a 21-year-old young male with fever, vomiting, and pain in the abdomen who was diagnosed with acute pancreatitis as a complication of dengue infection. We highlight the importance of screening for acute pancreatitis in patients with dengue presenting with pain in the abdomen as it may be a rare but important complication of dengue fever.

## Introduction

Dengue is a common viral infection seen in tropical areas. It is caused by the dengue virus belonging to the *Flavivirus* genus. Dengue virus has four serotypes, and it spreads through mosquito bites (*Aedes aegypti* and albopictus). It is endemic in tropical and subtropical regions. Dengue hemorrhagic fever (DHF) and dengue shock syndrome (DSS) are both dangerous conditions that can prove to be fatal due to plasma leakage, fluid buildup, respiratory difficulties, severe bleeding, or organ dysfunction [1]. Common severe dengue sequelae include myocarditis, encephalitis, acute-onset weakness of the motor system, Guillain-Barré syndrome, fulminant hepatic failure, lupus erythematosus, hemophagocytic syndrome, and acute-onset renal failure [2,3]. Acute pancreatitis, however, is an uncommon consequence of dengue fever and is mostly associated with severe dengue hemorrhagic fever.

## Case presentation

A 21-year-old male patient came to the hospital with complaints of fever, vomiting, pain in the abdomen, headache, and myalgia for three days. The patient was apparently alright three days ago when he started complaining of fever that was sudden in onset, intermittent, without diurnal variation, and not associated with chills or rigors. The fever was temporarily relived with antipyretics. He also had generalized weakness and joint pain associated with fever.

The patient also complained of pain in the abdomen for three days. The pain was diffuse, present in the epigastrium and umbilical region, and aggravated after consumption of food. It was associated with nausea and vomiting. The patient had around four to five episodes of vomiting after food every day. Vomitus contained food particles. There was no hematemesis. The patient was nonalcoholic.

On examination, his pulse rate was 88 beats per minute, blood pressure was 110/80 mmHg in the right arm in the supine position, and oxygen saturation was 98% on room air. On abdominal examination, there was tenderness in the umbilical area. Heart sounds were normal, no murmur was heard, normal breath sounds were heard, and there was no focal neurological deficit. 

On investigation, the patient tested positive for nonstructural antigen 1 for dengue fever. Platelets on admission were 53000/mm^3^, which gradually decreased until the fifth day after admission and then progressively increased (Table 1). The patient’s serum amylase and lipase were found to be raised.

**Table 1:** Laboratory results of the case

	1/8/21	3/8/21	4/8/21	5/8/21	6/8/21	7/8/21	8/8/21	9/8/21
White blood cell count (normal range: 4,000–11,000/mL)	3200/mm^3^	2600/mm^3^	2500/mm^3^	2200/mm^3^	1800/mm^3^	1600/mm^3^	3900/mm^3^	7800/mm^3^
Platelet count (normal range: 150,000–450,000/mL)	53,000/mm^3^	40,000/mm^3^	32,000/mm^3^	38,000/mm^3^	60,000/mm^3^	82,000/mm^3^	96,000/mm^3^	150,000/mm^3^
Serum amylase (normal range: 30–110 IU/L)		69 IU/L		268 IU/L	296 IU/L	325 IU/L	150 IU/L	90 IU/L
Serum lipase (normal range: 24–151 IU/L)		1590 IU/L		2760 IU/L	2890 IU/L	3250 IU/L	1000 IU/L	300 IU/L

**Figure 1:**
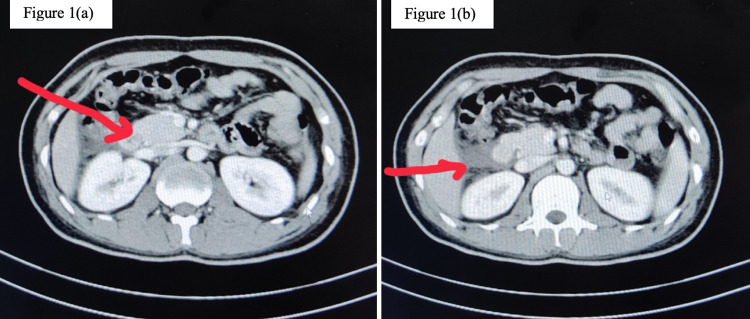
(a) Bulging of the uncinate process. (b) Peripancreatic fluid collection suggestive of acute pancreatitis.

Ultrasonography of the abdomen showed a bulky pancreas without ascites. Following this, contrast-enhanced computed tomography of the abdomen was done, which revealed bulging of the uncinate process of the pancreas and peripancreatic fluid collection along with minimal ascites (Figure 1). These features were suggestive of acute pancreatitis with a score of 2/10 (mild) as per the revised Atlanta classification of acute pancreatitis [4].

The patient was kept nil by mouth and was treated with intravenous fluids, antibiotics (ceftriaxone and doxycycline), antipyretics, opioids, and proton pump inhibitors. During the course of the hospital stay, the patient improved gradually along with an increase in platelet count. The patient did not require any platelet transfusion. Along with the improvement in hematological parameters, there was a decrease in amylase and lipase levels. He was discharged in stable condition eight days after admission in stable condition and is presently doing well on follow-up.

## Discussion

Acute pancreatitis is an inflammation of the pancreas most commonly caused by gallstones and excessive alcohol use. Infectious agents are also responsible for a few cases. Pancreatitis can be classified as (a) definite pancreatitis when a strong radiological or surgical confirmation is present, (b) probable pancreatitis when biochemical parameters with more than three times increase in serum amylase or lipase along with symptoms that are characteristics of pancreatitis are confirmed, and (c) possible pancreatitis in the presence of only biochemical confirmation with no symptoms suggestive of pancreatitis [5]. The patient had definite pancreatitis according to these criteria since he had radiological evidence for it. Dengue fever is caused by the dengue virus, which has four different antigenically diverse serotypes, namely, DEN-1, DEN-2, DEN-3, and DEN-4, which are all members of the *Flaviviridae* family [6]. Dengue virus is spread from person to person by mosquito bites from the *Aedes aegypti* and *Aedes albopictus* species. Infections range from asymptomatic to severe. Classic dengue fever (DF) or dengue hemorrhagic fever (DHF) are symptomatic diseases that may or may not be associated with shock [7,8].

DF affects people of all ages and genders, with children having a milder course than adults. Classic DF symptoms include sudden onset of high-grade fever with chills, intense headache, muscle and joint pain, retro-orbital pain, anorexia, generalized weakness, abdominal pain, dragging pain in the inguinal region, sore throat, and general depression after an average incubation period of five to six days. Fever is usually followed by remission for a few hours to two days; however, this is not always the case (biphasic curve). In 80% of patients, skin eruptions emerge following remission or the second febrile episode, which lasts for one to two days [5].

Pancreatitis with atypical presentations in the form of coinfection with typhoid and dengue have been reported before [9]. In dengue fever, the specific pathophysiology of pancreatic involvement remains unknown. However, one postulate suggests that it could be because of direct virus invasion leading to swelling and wrecking of acinar cells of the pancreas; another postulate suggests that shock, which occurs as a result of dengue shock syndrome, may damage the pancreas or cause an acute infection that leads to autoimmune feedback against islet cells of the pancreas and causes edema of the ampulla of Vater, obstructing pancreatic fluid outflow [10]. It is essential to detect pancreatitis at an early stage to prevent life chronic and fatal complications of pancreatitis [11].

Abdominal discomfort is a usual complaint of patients with dengue fever (40%), and it is frequently related to dengue hemorrhagic fever. Acute hepatic inflammation, acute cholecystitis that can be acalculous, acute inflammation of the pancreas, and acute inflammation of the colon are some of the reasons for abdominal pain in dengue fever [5].

As the patient was thrombocytopenic and there was a possibility of developing acute hemorrhagic pancreatitis, the platelet count of the patient was regularly monitored. The patient’s platelet count increased gradually without the need for platelet transfusion. The patient was treated successfully with ceftriaxone, doxycycline, antiemetics, intravenous fluids, antipyretics, opioids, and proton pump inhibitors and ultimately made a full recovery. This was in contrast to cases reported by Simadibrata et al. and Kumar et al. where the patients had developed severe dengue hemorrhagic fever with acute pancreatitis [11,12]. Both these case reports showed complete recovery with the help of conservative management for acute pancreatitis, which was also followed in our case. In another study conducted by Setiawan et al., 142 patients who had dengue hemorrhagic fever with acute pancreatitis were evaluated; 72 had mild DHF (grade I or II), and 70 had severe DHF (grade III or IV) [13]. In this study, the patients were also screened for appearance seen on ultrasonography of the abdomen. Of the patients, 36 (25%) had hyperechoic pancreas, 98 (69%) had isoechoic pancreas, and eight (6%) had hypoechoic pancreas on ultrasonography.

Therefore, timely diagnosis and management, and proper monitoring prevented our patient from developing a hemorrhagic fever, which is otherwise well associated with acute pancreatitis seen in dengue infection.

## Conclusions

Dengue-induced pancreatitis is a complication that is lesser studied. Dengue can have complex manifestations in the form of thrombocytopenia, dengue hemorrhagic fever, and dengue shock syndrome. The development of acute pancreatitis further complicates the clinical spectrum and prognosis and management. Timely diagnosis and management of the dengue fever complicated by acute pancreatitis reduce mortality and morbidity such as in our case; hence, the treating physicians should be well aware of such deadly complications that may arise with otherwise benign-looking dengue fever.
